# Cross-cultural adaptation and psychometric evaluation of the Sinhala version of Lawton Instrumental Activities of Daily Living Scale

**DOI:** 10.1371/journal.pone.0199820

**Published:** 2018-06-28

**Authors:** Dhammika Deepani Siriwardhana, Kate Walters, Greta Rait, Juan Carlos Bazo-Alvarez, Manuj Chrishantha Weerasinghe

**Affiliations:** 1 Research Department of Primary Care and Population Health, University College London, London, United Kingdom; 2 Department of Disability Studies, Faculty of Medicine, University of Kelaniya, Ragama, Sri Lanka; 3 Centro de Estudios de Población, Universidad Católica los Ángeles de Chimbote (ULADECH-Católica), Chimbote, Peru; 4 Department of Community Medicine, Faculty of Medicine, University of Colombo, Colombo, Sri Lanka; University of New South Wales, AUSTRALIA

## Abstract

**Introduction:**

Instrumental activities of daily living (IADL) are cognitively complex activities related to independent living in the community. Robust IADL scales are needed, however the psychometric properties of instruments have been little evaluated. There is no validated instrument for Sri Lankan older populations. Sri Lanka has the highest proportion of older people in South Asia with rapid population ageing. Therefore, it is essential to have standard instruments to assess activity limitations. We aimed to cross-culturally adapt the original Lawton Instrumental Activities of Daily Living Scale from English to Sinhala and evaluate the psychometric properties of the Sinhala version.

**Methods:**

Cross-cultural adaptation of the instrument was performed. The instrument was validated in a sample of 702 community-dwelling older adults aged 60 years and above in Sri Lanka. Reliability (internal consistency and inter-rater reliability) was assessed. Construct validity of the scale was evaluated by performing exploratory and confirmatory factor analysis and testing convergent and divergent validity.

**Results:**

The Lawton IADL scale was successfully adapted to Sri Lankan context. Internal consistency of the scale was very high (Cronbach’s alpha = 0.91). Very good inter-rater reliability was observed with very good agreement for all items. Inter-class correlations for overall IADL score ranged from 0.57 to 0.91. Results of the exploratory and confirmatory factor analyses supported the unidimensionality of the scale. Goodness of fit indices in confirmatory factor analysis were in acceptable range (CFI = 0.98, SRMR = 0.06, NNFI = 0.97). Strength of associations were significant and in the expected direction. Results of the known group validity were also significant, confirming the convergent and divergent validity.

**Conclusion:**

The Lawton IADL scale was successfully translated and culturally adapted to Sinhala language. The Sinhala version demonstrated excellent reliability and construct validity. Given good psychometric properties, this scale would be recommended for use in future research.

## Introduction

‘Activities of daily living’ measurement instruments are commonly used to assess the activity limitations. Two types of activities are assessed; Basic Activities of Daily Living (BADL) and Instrumental Activities of Daily Living (IADL). BADL are cognitively less complex self-maintaining tasks which include feeding, dressing, bathing, toileting, etc. They do not require attentional processes. Conversely, IADL are more complex and require higher level cognitive functions such as memory, attention and executive functions [1, 2]. Example IADL tasks are food preparation, housekeeping tasks, taking own medication, handling finances etc. These activities are important to lead an independent life [3]. IADL limitations often present with mild cognitive impairment and early dementia [4].

A number of questionnaires are available to assess IADL [3, 5], however, no gold standard exists [5]. One of the most widely used is the Lawton Instrumental Activities of Daily Living Scale developed in 1969 [2, 6]. A few modifications to the original scale are also available in the literature; modified Lawton- Brody scale proposed in 1988 [7], Lawton IADL scale in MFA (Multidimentional Functional Assessment of Older Adults) [8] and Lawton IADL scale in MAI (Multilevel Assessment Instrument) [9]. At present no agreement on the quality of IADL questionnaires exists. Moreover, the psychometric properties of commonly used IADL questionnaires are either unavailable or do not meet the standard quality [3].

Cultural adaptability, reliability and validity of the original [10–13] and Lawton IADL scale in MAI [14] have been tested in older populations (aged ≥60 or ≥65 years) in studies conducted in Iran, Spain, Greece, Singapore and Hong Kong. Study populations included patients with dementia [10], outpatients of memory clinics [12], patients who attended emergency rooms in with a hip or wrist fracture due to a fall [11], institutionalized older adults [14] and community living older adults [13].

We found three studies reporting IADL in Sri Lankan older adults [15–17]. However, none of the studies reported use of standard questionnaire to assess IADL, and instead used a few selected IADL tasks. Only four IADL tasks have been assessed in two studies [15, 16] and six in the remaining study [17]. We could not identify any culturally adapted, psychometrically tested instrument available to assess instrumental activities of daily living in Sri Lanka. It is important to have a standard instrument for this purpose as Sri Lanka has the highest proportion of older adults among South Asian countries [18] and considered as one of the fastest ageing populations in the South East Asia [19]. IADL limitations are associated with both poor quality of life [20] and increased healthcare costs [21]. Understanding the current IADL limitations of older adults in Sri Lanka using a robust standardised measure will inform planning of health and social care services with anticipated rapid population ageing. Therefore, the objective of this study was to cross culturally adapt the original Lawton Instrumental Activities of Daily Living scale from English to Sinhala and to evaluate the psychometric properties of the Sinhala version.

## Materials and methods

**Fig 1:**
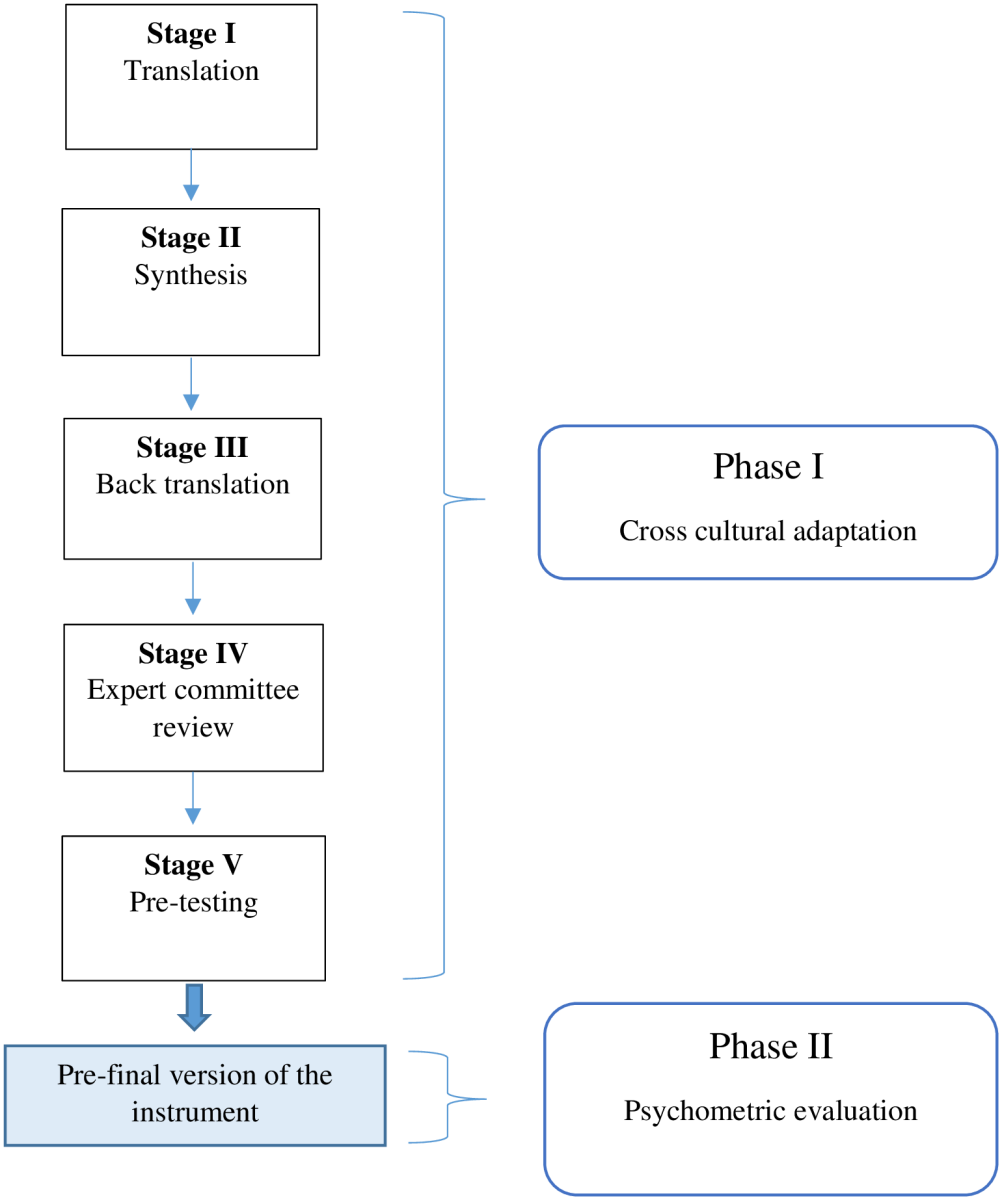
Study methodology.

The methodology of this study comprised of two phases. Phase one involved cross cultural adaptation of the Lawton IADL scale. Phase two was evaluating the psychometric properties of the scale which included testing the reliability (internal consistency and inter-rater reliability) and validity (cross-cultural validity, structural validity, convergent and divergent validity). Fig 1 illustrates the study methodology.

### Lawton Instrumental Activities of Daily Living Scale

We choose the original Lawton IADL scale for this study [2] (please see S1 File). It is a widely used instrument to measure IADL of older adults in different settings; community, clinics or hospitals [6]. Moreover, it is easy to administer (within 10–15 minutes). Most newer scales have also been derived from the original Lawton IADL scale [5]. The scale encompasses eight activities which includes ability to use telephone, shopping, food preparation, housekeeping, laundry, transport, ability to handle finances and responsibility for own medication. Each activity has varying number of response options indicating participant’s degree of ability to perform each activity starting from completely independent status to completely dependent status. Despite having number of responses under each activity, participants are classified into two categories as 1 (independent) and 0 (dependent) during the scoring. The total score of the scale ranges from 0 (dependent) to 8 (independent). Historically women were scored on all the items of the scale and men were scored for only five items of the scale excluding the food preparation, housekeeping and laundering activities. However, the current recommendation is to assess all activities with both sexes [22].

The original scale uses the self-repot/surrogate report ‘actual performance’ question stem, and later versions offered options of assessing self-repot/surrogate report ‘actual performance’ and ‘capacity’. We decided to use the self-reported ‘capacity’ question stem with the items and response structure of the original scale as in the Sri Lankan cultural context older adults are often supported by their own children and relatives. According to the recent census 17% of adults aged 60 years and above live with their own children [23]. Hence, some are not fully engaged doing certain IADL activities like housekeeping, shopping, preparing meals, handling finances even though they are fully capable of those. Sri Lanka is a country with good gender equality and we therefore used all the items in the scale with both males and females. Permission was granted from Oxford University Press to translate and republish the original scale in Sinhala language.

### Phase I- Cross cultural adaptation process

We used the systematic method proposed by Beaton and colleagues [24] for the cross cultural adaptation of self-reported measures.

Stage 1- Forward translation: Two independent bilingual translators who have a background in public health (DDS) and community medicine (MCW) translated the English version of entire instrument into Sinhala. The mother tongue of the both translators were Sinhala. They independently recorded the issues they had while translating the instrument.
Stage 2- Synthesis of the translations: A common Sinhala version of the instrument was created using the two independent translated versions.
Stage 3-Back translation: The synthesis version created at the second stage was used for back translation process. Two translators (TW, SJ different to stage 1 translators) who are fluent in both English and Sinhala languages conducted the back translations independently. Both were blind to the original instrument or original independent translated versions. Two back translated versions were compared with the original English version of the instrument for a validity check.
Stage 4- Expert committee review: A panel of experts from medical, allied health science, sociological backgrounds and translators (forward and backward) reviewed the two forward translations, two backward translations with the original scale. Consultations were conducted in person or using digital technology. MCW coordinated this stage. Semantic, idiomatic, experiential and conceptual equivalence of the instrument were discussed at these meetings. Any issues raised were addressed and a preliminary version of the instrument was created and circulated among the review members.
Stage 5- Pre-test: The preliminary version of the instrument was pre-tested with five male and female older adults in different age categories living in the district where the psychometric testing was planned. The pre-final version of the instrument was created to use in the psychometric evaluation.

### Phase II- Psychometric evaluation

#### Study design and participants

Psychometric evaluation; reliability and validity testing of the instrument was carried out as part of a large population based cross sectional study conducted in a district of Sri Lanka (Kegalle). The study population was community dwelling older adults, aged 60 years and above permanently residing in the rural sector of the district. Older adults who were unable to give the informed consent; older adults with severe dual hearing and vision impairment, aphasia following a stroke, severe stages of dementia, and those with unstable severe mental illnesses and terminally ill were excluded. The estimated sample size was 750 participants. Three-stage area probability sampling was utilized to recruit the participants. Fifty clusters were selected using probability proportionate to size technique covering entire district. Fifteen participants were recruited from each cluster based on the population demographics of rural sector of Sri Lanka [25]. According to the scale of sample size adequacy by Comrey and Lee (1992) sample of 500 considered as very good where 1000 or more considered as excellent in Exploratory Factor Analysis (EFA) [26] Five nursing graduates collected the data from the entire sample. They were given a comprehensive training on all aspects of the study. Participation for the study was voluntary and informed written consent was obtained from all the participants prior to collect data. The ethical clearance for this study was obtained from two ethics review committees at University College London (Project ID: 8155/001) and Faculty of Medicine, University of Colombo, Sri Lanka (Protocol No. EC-16-071).

#### Data analyses: Participants’ characteristics and distribution of Lawton IADL scale-Sinhala version scores

The characteristics of the study sample was described with descriptive statistics. The eight items of the scale were coded to preserve the original response structure as they do not have uniform response structure (ability to use telephone (1–4), shopping (1–4), food preparation (1–4), housekeeping (1–5), laundry (1–3), transport (1–5), responsibility for own medication (1–3) and ability to handle finances (1–3). The minimum number represents the response indicating complete dependent status for each item whilst the maximum number represent the response indicating highest independent status. However, when assigning scores according to the guidelines of the scale, response for each item was coded either as 0 (dependent) or 1 (independent). Hence, the total score of the scale ranges from 0 to 8.

#### Reliability testing

We assessed internal consistency (the extent to which different items measured the same construct [27, 28]) using standardized Cronbach’s alpha (as scale items do not have uniform response structure) and interpreted the same using the criteria proposed by Nunnally [29].

We assessed inter-rater reliability (IRR) [28] in a randomly selected 12% of the total sample (n = 89), representing 26 clusters. The number of participants recruited from each cluster varied from 1–5 with the modes of 3 and 4. Research assistants (5 raters) administered the IADL scale. After a gap of 2.5 to 3 hours the primary investigator (PI- DDS) re-administered the scale with the same participants. Therefore, each participant has been assessed by two raters (A/B/C/D/E and DDS).

We assessed the IRR of the each individual item considering its original response structure (ordinal) and after scoring (binary). Participants with missing values were excluded. For ordinal case, inter-rater reliability was calculated using unweighted percentage agreement coefficient, quadratic weighted Cohen’s kappa and Gwet’s AC_2_ with quadratic weights [30]. For binary case unweighted percentage agreement coefficient, Cohen’s kappa and Gwet’s AC_1_ were used. Both Gwet’s AC_1_ and AC_2_ agreement coefficients are corrected for chance agreement and adjusted for misclassification errors. Moreover, they are consistent with the percentage agreement [31]. Values of Cohen’s kappa, Gwet’s AC_1_ and AC_2_ were interpreted using criteria proposed by Landis and Koch [32]. Intra Class Correlation (ICC) was used to assess the agreement of the overall score of the scale between each rater and the PI. Single rating, absolute agreement, two way mixed effects model was used [33]. All the agreement coefficients and ICCs were computed using *kappaetc* user written Stata programme. Stata version 14 (StataCorp, College Station, Texas, USA) was used for the analyses. Guidelines for Reporting Reliability and Agreement Studies (GRRAS) proposed by Kottner et al were followed [34].

#### Validity testing

We assessed the construct validity of the IADL scale, including cross cultural validation (as above), structural validation (using factor analysis [28, 35]) and hypothesis testing [36] to establish the convergent and divergent validity of the scale [37, 38].

Exploratory factor analysis (EFA) explores the underlying factor structure of a construct [39, 40]. We performed EFA with 702 participants to test the hypothesis that the scale is unidimensional i.e. the 8 items in the scale represents one construct (instrumental activities of daily living). Original response structure of the scale was used in the analysis. Parallel analysis (PA) was run to determine the number of factors to retain in the model. PA was carried out on polychoric (two step) correlations with permuted samples, using principal component estimation and mean eigenvalue criterion [41]. Principal axis factoring was chosen as the factor extraction method because our data is ordinal and it violates the assumption of multivariate normality [40]. Principal axis factoring is also capable of detecting weak factors [42]. The Kaiser-Meyer-Olkin (KMO) statistic and Bartlett’s test of sphericity were used to determine the appropriateness of running the factor analysis. KMO values varies from 0 to 1 and values >0.5 are acceptable [43]. Bartlett’s test requires to yield significant result (p<0.05). Communalities ≥0.4 and factor loadings ≥ 0.5 were considered as satisfactory [39]. The analysis was performed on the polychoric (two step) correlations using SPSS R-menu v2.0 [44].

Confirmatory factor analysis (CFA) was performed to explore whether the observed data fit hypothesised factor structure of the IADL scale. Analysis was performed with the original response structure. To accommodate the ordinal response structure of the scale items, CFA was performed on asymptotic covariance matrix that calculated using the polychoric correlations. Diagonally weighted least square technique was used as the estimation method, recommended use when fitting structural equation model with ordinal variables [45]. Several goodness of fit indices were evaluated to determine the model fit. Evaluated fit indices include chi-square value (Satorra-Bentler scaled chi-square) with its degrees of freedom and associated p value, Relative/normed (χ^2^/df) chi-square, Root Mean Square Error of Approximation (RMSEA), Non-Normed Fit Index (NNFI)/ Tucker Lewis Index (TLI), Comparative Fit Index (CFI), Standardised Root Mean Residual (SRMR) and Parsimonious Normed Fit Index (PNFI). Insignificant chi square at a threshold of 0.05 is indicative of good model fit [46]. No consensus is available for the acceptable ratio of relative chi-square. Wheaton et al suggested a value of 5.0 [47]. For RMSEA Tuker and Lewis [48] suggested a cut-off of 0.06 whereas Steiger [49] proposed a strict upper limit as 0.07. For NNFI and CFI cut-off value of ≥ 0.95 accepted as good model fit [48, 50]. For SRMR a value of ≤ 0.08 considered as appropriate [50]. No threshold level has been specified for PNFI. CFA was performed on LISREL 9.30 student edition.

Historically Lawton et al (1969) proposed using the full scale (8 items) with females and five items (excluding food preparation, housekeeping and laundry) for males [2]. However, they had not checked the structural validity of IADL scale on this aspect. Therefore, we performed both EFA and CFA for females and males separately including all items.

The Barthel index of daily living measures the disability or dependence in basic activities of daily living, which are cognitively less complex tasks than IADL. [51]. Mild cognitive impairment is also associated with impairments in IADL [52]. Montreal cognitive assessment (MoCA) is a brief screening tool for mild cognitive impairment [53]. To assess the convergent validity, we hypothesised that IADL score is positively correlated with Barthel index score and MoCA score. Spearman’s correlation coefficient was used to quantify the magnitude of the correlation. We used following criteria to interpret the size of the correlation coefficients; (0 to ±0.3) negligible, (±0.3 to ±0.5) low, (±0.5 to ±0.7) moderate, (±0.7 to ±0.9) high and (±0.9 to 1.0) as very high correlation [54].

We used known group validity method to assess the divergent validity of the IADL scale. Advanced age is associated with the limitations of IADL [55]. Therefore, we hypothesised lower IADL scores for older age groups. The participants of this study were divided into two groups based on the median age of the sample. Median IADL score of the two groups were tested using Mann-Whitney U non-parametric test since our IADL score does not follow a normal distribution. The significance level was set as 0.05. Both analyses performed using IBM SPSS 24 software (SPSS INc., Chicago, IL, USA).

## Results

### Cross cultural adaptation of Lawton IADL scale

Stage 1- Forward translation was performed as planned. Both forward translators encountered following issues. The last response for the item 1- ‘Ability to use telephone’, in the original scale is ‘does not use telephone at all’. We felt that this response can be interpreted in different ways. A person could be not using a telephone at all since he/she does not have a one or incapable of using it. Incapability could be due to an impairment or the person has never used it before and have no skills to use it. The same issue was noted for the last response of item 6-‘Mode of transportation’. Both translators were uncertain about the identical Sinhala word to ‘instrumental’.
Stage 2- PI prepared the synthesis version with the aid of both Sinhala versions.
Stage 3- Backward translation was also carried out as planned. Both backward translated versions showed good agreement with the original English version.
Stage 4- By considering the issues raised in the forward translation process (stage 1), panel of experts agreed to replace the last response of item 1 with the meaning of ‘incapable of using the telephone at all’ in Sinhala. However, they acknowledged that still the response could not be applicable to a person who has never used a telephone. Hence, the suggestion was to ask whether they have ever used a telephone if their response is ‘incapable of using the telephone at all’ and make a note in the questionnaire. Similarly the last response for item 6 (‘does not travel at all’) was replaced from ‘incapable of travelling at all’. Example apparels used in the second response of item 5-‘Laundry’ were replaced from ‘socks’ and ‘stockings’ to ‘small hanker chief’ and ‘small towel’, as more relevant to Sri Lankan older adults living in tropical climate. Example activities used in the final item-‘ability to handle finances’ were ‘budgets, writes checks, pays rent, bills, goes to bank’. They were replaced from making a payment for electricity or water bill and doing bank transactions. We could not find an identical Sinhala word for the word ‘instrumental’. Therefore, we substituted that word to ‘non-basic’ in Sinhala.
Stage 5- No difficulties were encountered in pre-testing and the IADL Sinhala version and showed good acceptability.

### Psychometric evaluation of Lawton IADL scale-Sinhala version

#### Socio-demographic characteristics of the study participants

Data collection was conducted from 03^rd^ October to 23^rd^ December 2016. Seven hundred forty six participants were recruited for the study. Twenty three participants were excluded as they are not fully conversant in Sinhala language. Twenty one participants were excluded as they had never used a telephone and/or were completely unaware of how to cook. Five males and eight females had never used a telephone. Seven males were unaware of how to cook. One male participant was excluded for both reasons. Therefore, the effective sample was 702.

**Fig 2:**
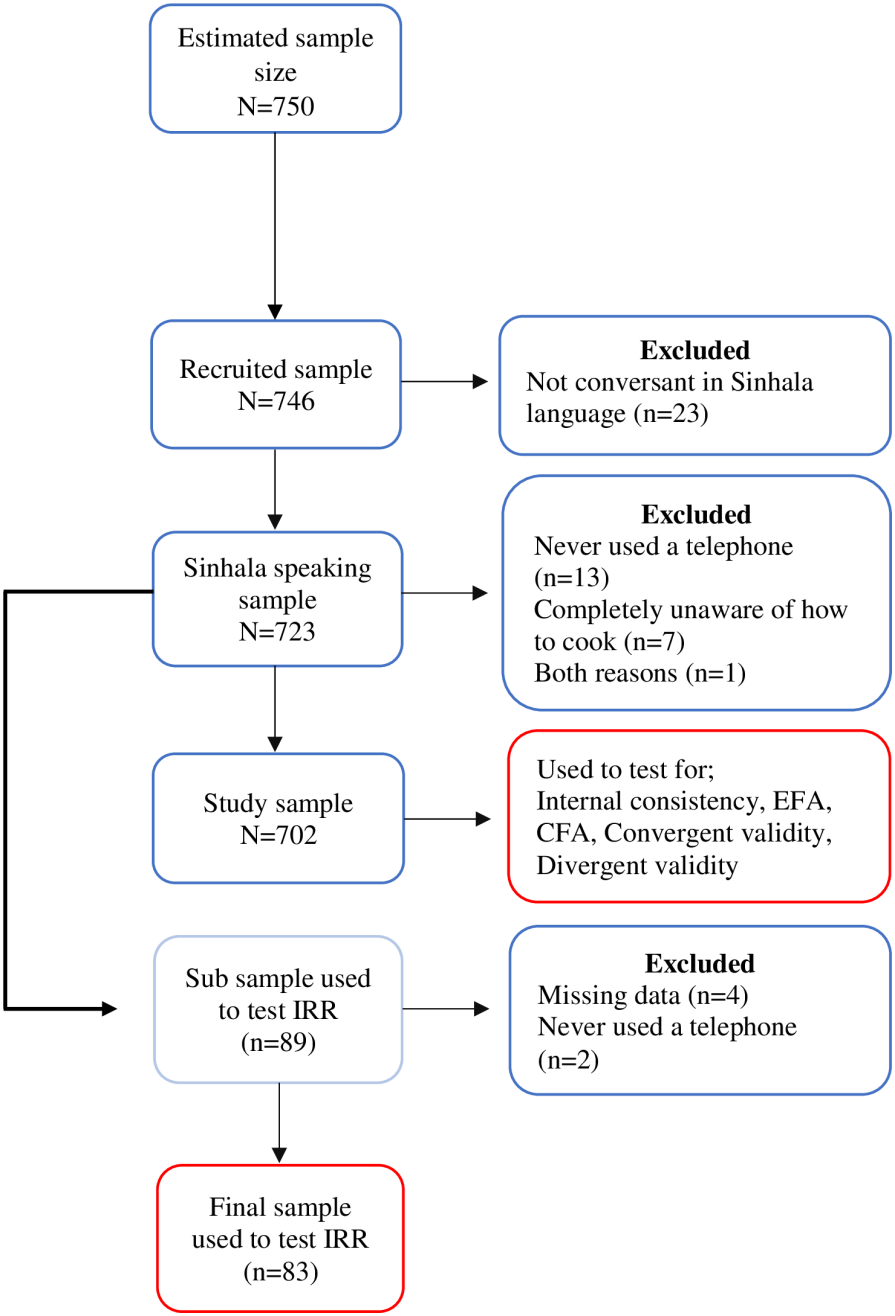
Study flow chart.

A sub sample from Sinhala speaking participants was invited to test the IRR. Six participants invited were excluded when testing for IRR. Of the six, two had not used a telephone ever and response for one item in the scale was missing for four participants in the PI’s dataset. Fig 2 demonstrates the study flow chart.

Table 1 presents the socio-demographic characteristics of study sample (n = 702) and the sub sample used to test IRR (n = 83). The percentage of the females in the study sample was 53.7%. The median (IQR) age of the sample was 67 (63, 75) years. The age of the participants ranged from 60 years to 94 years. The median (IQR) age of the sub sample used to test IRR was 68 (63, 73) years. The age of the participants was ranged from 60 years to 91 years.

**Table 1:** Socio-demographic characteristics of the study participants.

Characteristics		Study sample n (%)	Sub sample used to test IRR n (%)
**Sex**	Male	325 (46.3)	30 (36.1)
	Female	337 (53.7)	53 (63.9)
**Age category (years)**			
	60–64	238 (33.9)	28 (33.7)
	65–69	189 (26.9)	22 (26.5)
	70–74	91 (13.0)	15 (18.1)
	75–79	91 (13.0)	6 (7.2)
	≥80	93 (13.2)	12 (14.5)
**Marital status**			
	Never married	33 (4.7)	7 (8.4)
	Married	427 (60.8)	43 (51.8)
	Separated	12 (1.7)	2 (2.4)
	Divorced	5 (0.7)	1 (1.2)
	Widowed	223 (31.8)	30 (36.2)
	Cohabiting	2 (0.3)	-
**Living arrangement**			
	With spouse	79 (11.3)	11 (13.3)
	With children/other family	580 (82.6)	65 (78.3)
	Alone	43 (6.1)	7 (8.4)
**Educational status**			
	Never schooled; unable to read and write	31 (4.4)	2 (2.4)
	Never schooled; able to read and write	3 (0.4)	1 (1.2)
	Passed Grade 1–5 (1–5 years)	163 (23.2)	22 (26.5)
	Passed Grade 6–10 (6–10 years)	246 (35.0)	29 (35.0)
	Passed G.C.E. O/L (11 years)	181 (25.8)	24 (28.9)
	Passed G.C.E. A/L (13 years)	60 (8.6)	5 (6.0)
	Higher education (16 years minimum)	18 (2.6)	-
Perceived financial status*			
	Finding it difficult/very difficult	140 (20.0)	15 (18.1)
	Just about getting by	380 (54.1)	48 (57.8)
	Living comfortably	182 (25.9)	20 (24.1)

*Using a question of Weich and Lewis (1998) (Weich S, Lewis G. Poverty, unemployment, and common mental disorders: population based cohort study. BMJ. 1998;317(7151):115.)

#### Distribution of Lawton IADL scale-Sinhala version scores

The frequency distribution of the responses for each item and overall score are presented in S1 and S2 Figs respectively. A negatively skewed distribution was observed for responses of all the items and overall score. Table 2 presents the median and inter quartile range for the scores of each item. None of the items’ or total score distributed normally.

**Table 2:** Item descriptive statistics of IADL scale-Sinhala version.

Item	Item description	Min, Max	Median (IQR)	Dependent n (%)	Independent n (%)
Item 1	Ability to use telephone	1,4	4 (2,4)	73 (10.4)	629 (89.6)
Item 2	Shopping	1,4	4 (4,4)	146 (20.8)	556 (79.2)
Item 3	Food preparation	1,4	4 (4,4)	139 (19.8)	563 (80.2)
Item 4	Housekeeping	1,5	5 (5,5)	39 (5.6)	663 (94.4)
Item 5	Laundry	1,3	3 (3,3)	36 (5.1)	666 (94.9)
Item 6	Mode of transportation	1,5	5 (5,5)	56 (8.0)	646 (92.0)
Item 7	Responsibility of own medication	1,3	3 (3,3)	90 (12.8)	612 (87.2)
Item 8	Ability to handle finances	1,3	3 (2,3)	55 (7.8)	647 (92.2)
	Overall IADL score	0,8	8 (7,8)		

IQR- Inter quartile range

#### Internal consistency and Inter-rater reliability

The internal consistency of the scale with 8 items assessed by Cronbach’s alpha was 0.918. Cronbach’s alpha if an item deleted is presented in the Table 3.

**Table 3:** Cronbach’s alpha if an item deleted in IADL scale-Sinhala version.

Item	Item description	Cronbach’s alpha if an item deleted
Item 1	Ability to use telephone	0.928
Item 2	Shopping	0.899
Item 3	Food preparation	0.910
Item 4	Housekeeping	0.897
Item 5	Laundry	0.907
Item 6	Mode of transportation	0.902
Item 7	Responsibility of own medication	0.908
Item 8	Ability to handle finances	0.903

Table 4 presents the unweighted percentage agreement coefficient, weighted Cohen’s kappa and Gwet’s AC_2_ agreement coefficient for each item according to the responses in the original scale. Relatively low absolute percentage agreement was observed for the ‘Housekeeping’ item between PI and all five raters compared with the other items. However, for all the items primary investigator had a satisfactory absolute/unweighted percentage agreement coefficient ranged from 0.62 to1.00, poor to excellent weighted Cohen’s kappa (0.00 to 1.00) and substantial to almost perfect Gwet’s AC_2_ (0.62 to 1.00) agreement coefficient with all raters. Interestingly, weighted Cohen’s kappa was not computed when the percentage agreement too high or too low indicating the ‘kappa paradox’[56]. Please refer S1 Table for IRR for each item after scoring as binary. Rater A, C, and D showed ICC values above 0.8 indicating an excellent reliability. In overall, ICC values for all the raters were above 0.5 and indicate moderate to excellent reliability [33].

**Table 4:** Item wise inter-rater reliability with original response structure for Lawton IADL scale-Sinhala version and ICC for overall IADL score.

Item	Item description	PI-A (n = 13)			PI-B (n = 15)			PI-C (n = 17)			PI-D (n = 17)			PI-E (n = 21)		
		p	*κ*_w_	Gwet’s AC_2_	p	*κ*_w_	Gwet’s AC_2_	p	*κ*_w_	Gwet’s AC_2_	p	*κ*_w_	Gwet’s AC_2_	p	*κ*_w_	Gwet’s AC_2_
Item 1	Ability to use telephone	0.92	0.77	0.87	0.93	0.81	0.90	0.94	0.97	0.98	0.88	0.96	0.96	0.90	0.72	0.92
Item 2	Shopping	0.92	0.75	0.89	0.73	0.70	0.92	0.71	0.50	0.87	0.88	0.93	0.97	0.90	**-0.05**	0.90
Item 3	Food preparation	0.85	0.82	0.93	0.67	**0.00**	0.70	0.71	0.72	0.86	0.82	**0.00**	0.89	0.95	0.64	0.94
Item 4	Housekeeping	0.62	0.32	0.89	0.67	0.65	0.88	0.71	0.51	0.84	0.71	0.20	0.82	0.76	**0.00**	0.91
Item 5	Laundry	Not computed^‡^			0.80	**0.51**	0.85	0.88	0.77	0.96	0.82	**-0.06**	0.89	0.81	**0.00**	0.92
Item 6	Mode of transportation	0.77	0.85	0.88	0.73	0.62	0.81	0.76	0.82	0.89	0.82	0.84	0.92	0.90	**0.48**	0.87
Item 7	Responsibility of own medication	0.92	0.78	0.97	0.93	0.78	0.98	0.94	0.91	0.98	1.00	1.00	1.00	0.95	0.79	0.98
Item 8	Ability to handle finances	0.77	**0.41**	0.62	0.67	0.46	0.84	0.82	0.78	0.91	0.65	0.63	0.81	0.76	0.46	0.91
	**Intra Class Correlation** **(95% CI)**	0.91 (0.74, 0.97)			0.62 (0.20, 0.85)			0.89 (0.73, 0.96)			0.88 (0.64, 0.96)			0.57 (0.20, 0.80)		

**p**- Unweighted percentage agreement coefficient, ***κ***_**w**_—Cohen’s weighted kappa. Non-significant agreement coefficients (p>0.05) and zero agreement coefficients are displayed in bold.

Not computed^‡^, since ratings do not vary.

#### Exploratory factor analysis

**Fig 3:**
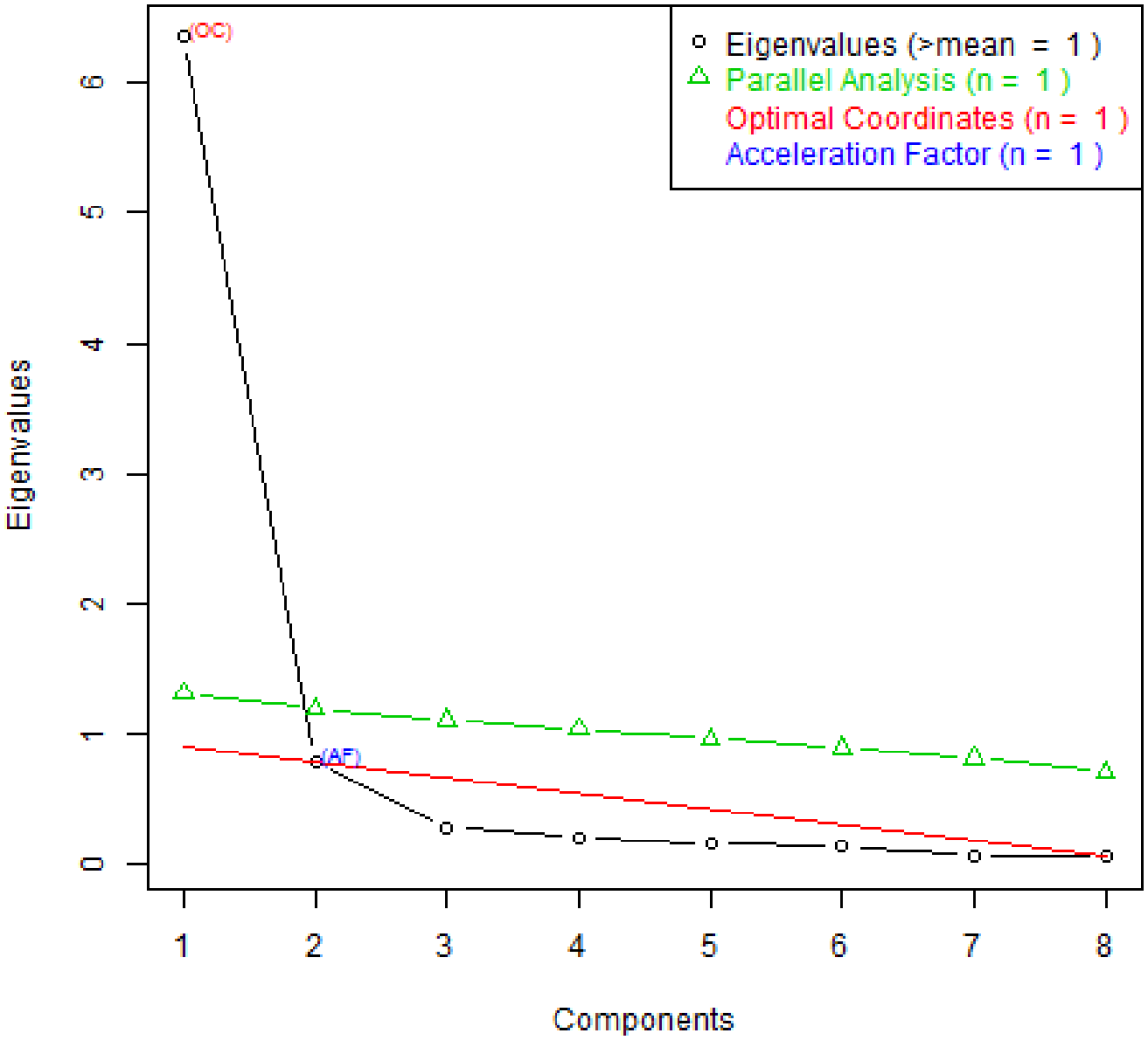
Parallel analysis based on permuted data.

Kaiser-Meyer-Olkin measure of sampling adequacy was 0.898 which is considered a ‘very good’ value [57]. The Significance value of Bartlett’s test of sphericity was <0.001, indicating that the correlations between the items were significantly different from zero. Results of the parallel analysis suggested to extract one factor (Fig 3), indicating the unidimensionality of the scale. The first factor explained 79.4% of the total variance. As shown in Table 5, the communalities of 8 items varied from 0.392 to 0.903 and factor loadings were varied from 0.626 to 0.950. Item scale correlation (corrected) for all the items were above 0.7 except for item 1. The polychoric (two step) correlations matrix is available in S2 Table.

**Table 5:** Results of the exploratory factor analysis.

Items	Item description	Exploratory factor analysis*		
		Communality	Factor loading	Item-scale correlation^†^
Item 1	Ability to use telephone	0.392	0.626	0.503
Item 2	Shopping	0.892	0.944	0.865
Item 3	Food preparation	0.724	0.851	0.724
Item 4	Housekeeping	0.903	0.950	0.883
Item 5	Laundry	0.782	0.884	0.771
Item 6	Mode of transportation	0.848	0.921	0.819
Item 7	Responsibility of own medication	0.787	0.887	0.745
Item 8	Ability to handle finances	0.819	0.905	0.825

^†^Item total correlation with its own Lawton IADL scale corrected for overlap.

*Fit indices: GFI (ULS) = 0.980, RMSR = 0.063

EFA results by sex also showed a stable item structure (8 items) across both females and males. Parallel analysis suggested to extract one factor in both cases (S3 and S4 Figs). The percentage of variance explained by the first factor was 80.2% for females and 81.5% for males. The communalities of 8 items varied from 0.357 to 0.934 and 0.421 to 0.925 for females and males respectively. The factor loadings were varied from 0.598 to 0.966 for females and from 0.649 to 0.962 for males. The polychoric (two step) correlations matrixes by sex are available in S3 and S4 Tables. Please refer S5 Table for additional results.

#### Confirmatory factor analysis

CFA results are presented in Table 6. The measurement model with standard factor loadings and uniquenesses are presented in S5 Fig. Standardized factor loadings were ranged from 0.660 to 0.958. Values of goodness of fit indices; NNFI, CFI and SRMR were in acceptable range indicating an excellent model fit. However, the chi-square value was significant, χ^2^ (20, 702) = 144.42, p<0.001. The value of relative chi-square (χ^2^/df) was 7.22 and not in the acceptable range. Similarly, RMSEA value was too high and neither in acceptable range.

**Table 6:** Results of confirmatory factor analysis.

Items	Item description	Confirmatory factor analysis^‡^	
		Standardized factor loading	Standard error
Item 1	Ability to use telephone	0.660	0.034
Item 2	Shopping	0.938	0.012
Item 3	Food preparation	0.871	0.021
Item 4	Housekeeping	0.958	0.008
Item 5	Laundry	0.926	0.017
Item 6	Mode of transportation	0.911	0.014
Item 7	Responsibility of own medication	0.873	0.023
Item 8	Ability to handle finances	0.918	0.013

^‡^ Fit indices: RMSEA (90% CI) = 0.283 (0.270–0.297), NNFI/TLI = 0.977, CFI = 0.984, SRMR = 0.06, PNFI = 0.701

In CFA by sex, standardized factor loadings were ranged from 0.645 to 0.973 and from 0.673 to 0.981 for females and males respectively. All the goodness of fit indices except chi-square, and RMSEA were in the acceptable range for both sexes. Please refer S6 and S7 Tables for additional results.

Item 1 (ability to use telephone) consistently demonstrated low communality, factor loading and item-scale correlation in EFA and low standardized factor loading in CFA. Furthermore, Cronbach’s alpha was slightly higher when item 1 was deleted from the scale (see Table 3). This finding was consistent even in the sex stratified analysis.

#### Convergent validity

The Spearman’s correlation coefficients between Lawton IADL score and the scores of Barthel index and MoCA were 0.61 and 0.41, indicating a moderate and low strength of association respectively. Both correlation coefficients were significant at p<0.001.

#### Divergent validity

The median age of the sample was 67 years. Therefore, the sample divided into two groups as age ≤67 years and >68 years. The median (IQR) IADL scores for both groups were 8 (0) and 7 (1) (Mann-Whitney U = 37,974, p<0.001) demonstrating a lower IADL score in older group as hypothesised.

## Discussion

### Summary of main findings

The Lawton Instrumental Activities Daily Living scale was successfully translated and culturally adapted to Sri Lankan context. The Sinhala version of the scale demonstrated excellent reliability and construct validity. Internal consistency of the scale was very high. Satisfactory agreement was observed between PI and the raters for all the items in the scale. With regard to the overall score, ICC values were between 0.57 and 0.91 which is indicative of moderate to very good agreement. Findings of EFA and CFA strongly supported the unidimensionality of the scale. In EFA, communalities and factor loadings for all the items were well above the cut-off values. Similarly all the goodness of fit indices in CFA were in the acceptable range except chi-square and RMSEA. Eight item structure scale was stable across both females and males. Results of the sex stratified EFA and CFA were also consistent with the main analysis. We observed moderate and low positive correlations between IADL score and scores of Barthel index and MoCA respectively. The scale was capable of detecting the difference of overall IADL score between age groups.

### Reliability

In line with other studies, Lawton IADL-Sinhala version has demonstrated an excellent internal consistency. Cronbach’s alpha coefficient was 0.91. Of all studies Spanish version has demonstrated the highest alpha value of 0.94 [11] whilst 0.86, 0.84 in Hong Kong Chinese (Lawton IADL-CV) and Greek versions respectively [12, 14]. The lowest was observed in the Persian version (Lawton IADL-PV) [10].

We found ICC values ranging from 0.57 to 0.91 for five raters, with three raters having values above 0.8, in a relatively large sample of 83 participants. The inter-rater reliability of the original scale was 0.85, however this study was with a small sample (n = 12), interviewed by one interviewer in the presence of the second rater who did not participate in the interviewing process [2]. Two further studies have reported the inter-rater reliability, with ICCs of 0.96 [10] and 0.99 [14] in similarly small studies. In the latter, inter-rater reliability was assessed with 9 participants on video-taped IADL abilities, and this method (videos) has been shown to produce higher inter-rater reliability [58].

Unlike our study, none of the prior studies have reported the item-wise inter-rater reliability of the scale. In those studies, ICC was computed based on the total score of the scale. It does not reflect how each rater marked the response for each item based on participant’s response.

### Validity

EFA results of our study strongly supported the unidimensionality of the scale and corroborate with the existing literature [11, 14]. In our study first factor explained 79.4% variance whilst 70.6% and 50.1% variances explained by the Hong Kong Chinese and Spanish versions correspondingly. Eight item structure has an excellent factorial validity across both sexes [11]. We excluded 8 male participants from the analysis since they were completely unaware of how to cook. However, this was only 2.4% of total males in the sample. In contrast, Ng et al (2006) found two strong factors underlying physical and cognitive domains of IADL in a multi-ethnic Asian population in Singapore [13]. Two factors explained 87.5% of variance. Interestingly, physical IADL domain included 5 items (grocery shopping, getting to places outside the house, doing housework/handyman work, doing laundry and preparing meals) and cognitive domain included remaining three items (using the telephone, taking medications and managing finances).

All the reported goodness of fit indices of CFA (RMSEA, TLI, and CFI) were satisfactory and all factor loadings were significant in the Spanish version [11]. Similarly all the factor loadings were significant in Sinhala version and values of TLI, CFI, and SRMR were in the acceptable range. However, our RMSEA value was not in the acceptable range. One possible explanation could be use of diagonally weighted least square technique estimation. Nye et al showed that RMSEA appears to be affected by sample size [59]. With a sample of 400, they have observed increase of cut-off value for RMSEA whilst SRMR seems to be performing relatively well which is similar to our case. While the chi-square value of our model was significant, this may often be the case with large sample sizes [60] and when data deviate from multivariate normality [61].

We observed a substantially low communality and factor loading for the first item; ‘ability to use the telephone’ in EFA and relatively low standardized factor loading in CFA. Item one has demonstrated a relatively low inter-item correlation with item 3, 4, and 5. This pattern was consistent across both sexes. Interestingly, we observed the same results for EFA with the Spanish version [11]. A possible reason could be transition of use of land/fixed telephones to the mobile devices. At present, most of the households in Sri Lanka use mobile phones. According to the statistics of Telecommunications Regulatory Commission of Sri Lanka, 12.1 and 122 fixed access and cellular mobile subscriptions per 100 inhabitants were reported respectively in 2016 [62]. Using a telephone does not only require a reasonable cognitive function. It is also affected by the sensory function and fine motor skills particularly when using the mobile phones. Therefore, the patterns of activity limitations assessing here is slightly different to other IADLs. However, it is still measuring something different than the other items as it represents the ability to communicate with the outside world, an important part of ageing well.

The Spearman’s correlation coefficient between the scores of Barthel index and Lawton IADL Spanish version was above 0.4 and mean IADL score was significantly different by age [11]. In our study, value of the same correlation was 0.61 and the median IADL scores were significantly different by age. Known group validity results of the Singapore study has also shown significantly different mean IADL scores across different age groups and gradual decrease of the mean IADL score values with increasing age [13]. In line with our findings studies conducted in Greece and Hong Kong also supported the convergent and/or divergent validity of the Lawton IADL scale by means of the strength of associations or known group validity [12, 14].

### Strengths of the study

The main strengths of this study were following a comprehensive and rigorous methodology, and using advanced statistical techniques to address the structure and distribution of the data. We performed the psychometric evaluation with a large random sample of Sinhala speaking community dwelling rural older adults. According to the recent census, 99% of Sri Lankan older adults live in the community [23]. In the original validation study, Lawton and Brody (1969) had not explored the factor structure of the IADL scale [2]. Therefore, we performed both EFA and CFA. We adhered to set of guidelines and best practices available in the literature when performing and reporting cross cultural adaptation of instrument, reliability testing, EFA and CFA [24, 33, 34, 39, 63, 64].

### Study limitations and recommendations

We excluded 21 participants (<2%) from the study population who reported they had never used a telephone or were completely unaware of how to cook. We could not assess the test-retest reliability and responsiveness of the scale due to inadequate resources available. The limitation of the scale itself is absence of a reference point of time. However, no guidelines exists as to the appropriate choice of reference point of time either [5].

We used the participant’s self-reported capacity of performing each activity in our Lawton IADL-Sinhala version as a measure of self-reported efficacy or capacity in performing activities. Some people may over or underestimate their true capacity and this may therefore not reflect the actual performance of these activities. Alternatively a researcher can also use the self-reported ‘actual performance’ question stem and make notes about not applicable items (where the participant may be capable but does not regularly perform the activity). In our study we used an interviewer-administered questionnaire with the respondent only. In future research self-reported and a key informant reported abilities of performing IADL tasks could also be compared. We have also not specified a reference point of time, instead the scale asks the general ability of performing each activity in day-to-day life.

## Conclusions

The Lawton IADL scale was successfully translated and culturally adapted to Sinhala language. The Sinhala version demonstrated an excellent reliability and construct validity with a large representative sample of Sinhala speaking community dwelling older adults. Given good psychometric properties, it is recommended to monitor the limitations of instrumental activities of daily living of community dwelling older adults in Sri Lanka. Lawton IADL-Sinhala version can be found in S2 File.

## Supporting information

S1 File The Lawton Instrumental Activities of Daily Living (IADL) Scale. (PDF)

S2 File The Lawton Instrumental Activities of Daily Living (IADL) Scale-Sinhala version. (PDF)

S1 Fig The frequency distribution of the responses for each item of the Lawton IADL-Sinhala version. (PDF)

S2 Fig The frequency distribution of the overall Lawton IADL score-Sinhala version. (PDF)

S3 Fig Parallel analysis results for females. (PDF)

S4 Fig Parallel analysis results for males. (PDF)

S5 Fig Confirmatory factor analysis model with standardized factor loadings. (PDF)

S1 Table Item wise inter-rater reliability when original responses for Lawton IADL-Sinhala version coded as binary. (PDF)

S2 Table Polychoric (two step) correlation matrix used in EFA (entire sample). (PDF)

S3 Table Polychoric (two step) correlation matrix used in EFA for females. (PDF)

S4 Table Polychoric (two step) correlation matrix used in EFA for males. (PDF)

S5 Table Results of exploratory factor analysis by sex. (PDF)

S6 Table Results of the confirmatory factor analysis by sex. (PDF)

S7 Table Goodness of fit indices of confirmatory factor analysis by sex. (PDF)
