# Supplementary material for: Cross-cultural adaptation and psychometric evaluation of the Sinhala version of Lawton Instrumental Activities of Daily Living Scale
Source: PLoS One. 2018 Jun 28;13(6):e0199820. doi: 10.1371/journal.pone.0199820 (PMC6023108; doi:10.1371/journal.pone.0199820)
Supplement: S1 File — (PDF) [file pone.0199820.s001.pdf]

S1 File. The Lawton Instrumental Activities of Daily Living (IADL) Scale.

**A. Ability to Use Telephone**	
1. Operates telephone on own initiative-looks up and dials numbers, etc.	1
2. Dials a few well-known numbers.	1
3. Answers telephone, but does not dial.	1
4. Does not use telephone at all.	0
**B. Shopping**	
1. Takes care of all shopping needs independently.	1
2. Shops independently for small purchases.	0
3. Needs to be accompanied on any shopping trip.	0
4. Completely unable to shop.	0
**C. Food Preparation**	
1. Plans, prepares, and serves adequate meals independently.	1
2. Prepares adequate meals if supplied with ingredients.	0
3. Heats and serves prepared meals or prepares meals but does not maintain adequate diet.	0
4. Needs to have meals prepared and served.	0
**D. Housekeeping**	
1. Maintains house alone or with occasional assistance (e.g., “heavy work-domestic help”).	1
2. Performs light daily tasks such as dishwashing, bed making.	1
3. Performs light daily tasks, but cannot maintain acceptable level of cleanliness.	1
4. Needs help with all home maintenance tasks.	1
5. Does not participate in any housekeeping tasks.	0
**E. Laundry**	
1. Does personal laundry completely.	1
2. Launders small items, rinses socks, stockings, etc.	1
3. All laundry must be done by others.	0
**F. Mode of Transportation**	
1. Travels independently on public transportation or drives own car.	1
2. Arranges own travel via taxi, but does not otherwise use public transportation.	1
3. Travels on public transportation when assisted or accompanied by another.	1
4. Travel limited to taxi or automobile with assistance of another.	0
5. Does not travel at all.	0
**G. Responsibility for own Medications**	
1. Is responsible for taking medication in correct dosages at correct time.	1
2. Takes responsibility if medication is prepared in advance in separate dosages.	0
3. Is not capable of dispensing own medication.	0
**H. Ability to Handle Finances**	
1. Manages financial matters independently (budgets, writes checks, pays rent, bills, goes to bank), collects and keeps track of income	1
2. Manages day-to-day purchases, but needs help with banking, major purchases, etc.	1
3. Incapable of handling money.	0

Lawton MP, Brody EM; Assessment of Older People: Self-Maintaining and Instrumental Activities of Daily Living, The Gerontologist 1969; 9 (3_Part_1): 179–186, doi:10.1093/geront/9.3_Part_1.179.
Reproduced by permission of Oxford University Press on behalf of The Gerontological Society of America. © 1969 The Gerontological Society of America. All rights reserved. For permissions please email journals.permissions@oup.com. Please visit: https://academic.oup.com/gerontologist/article/9/3_Part_1/179/552574
This scale is not included under the Creative Commons license of this publication.
