# Supplementary material for: Cross-cultural adaptation and psychometric evaluation of the Sinhala version of Lawton Instrumental Activities of Daily Living Scale
Source: PLoS One. 2018 Jun 28;13(6):e0199820. doi: 10.1371/journal.pone.0199820 (PMC6023108; doi:10.1371/journal.pone.0199820)
Supplement: S2 File — (PDF) [file pone.0199820.s002.pdf]

S2 File. The Lawton Instrumental Activities of Daily Living Scale- Sinhala version.

The Lawton Instrumental Activities of Daily Living (IADL) Scale- Sinhala version

ප්‍රාථමික නොවන දෛනික වැඩ කටයුතු කර ගැනීමේ හැකියාව මැන බැලීමේ ප්‍රශ්නාවලිය

උපදෙස්: සමීක්ෂණයට සහභාගී වන පුද්ගලයාගෙන්, පහත සඳහන් කාර්යයන් එදිනෙදා කිරීමට ඇති හැකියාව විමසා බලන්න. ඔහුට/ඇයට වඩාත් අදාල වන පිළිතුර ඉදිරියෙන් ඇති ලකුණ රවුම් කරන්න.

A. දුරකථනය භාවිත කිරීමේ හැකියාව

තනිවම දුරකථනය භාවිතා කළ හැක; ඕනෑම දුරකථන අංකයකට ඇමතුමක් ලබා ගත හැක. 1

හොඳින්ම දන්නා දුරකථන අංක කීපයකට පමණක් ඇමතුම් ලබා ගත හැක. 1

දුරකථනයට ලැබෙන ඇමතුම් වලට පිළිතුරු දීමට හැකි වුවත් තනිවම දුරකථන ඇමතුමක් ලබා ගැනීමට නොහැක. 1

දුරකථනය කිසිසේත්ම භාවිතා කළ නොහැක. 0

B. කඩයකට ගොස් නිවසට අවශ්‍ය භාණ්ඩ මිලදී ගැනීමේ හැකියාව

තනිවම කඩයකට ගොස් නිවසට අවශ්‍ය ඕනෑම දෙයක් මිලදී ගත හැක. 1

තනිවම කඩයකට ගොස් නිවසට අවශ්‍ය සුළු දෙයක් මිලදී ගත හැක. 0

කඩයකින් බඩු ගැනීමට යාමේදී වෙනත් අයකුද තමා සමඟ යා යුතුය. 0

භාණ්ඩ මිලදී ගැනීමට යාමට කිසිසේත්ම නොහැක. 0

C. ආහාර පිළියෙල කිරීම

ස්වාධීනව ප්‍රමාණවත් ආහාර වේලක් සැලසුම් කර, පිළියෙල කර, පරිභෝජනය දක්වා සකස් කර ගත හැක. 1

අවශ්‍ය කරන අමුද්‍රව්‍ය ලබා දුන්නහොත් ප්‍රමාණවත් ආහාර වේලක් පිළියෙල කළ හැක. 0

ආහාර රත් කිරීමට, පරිභෝජනය දක්වා සකස් කර ගැනීමට හැකි වුවත් ප්‍රමාණවත් ආහාර වේලක් පිළියෙල කළ නොහැක. 0

වෙනත් කෙනෙකු විසින් ආහාර පිළියෙල කර පිළිගැන්විය යුතුයි. 0

D. **ගේ දොර වැඩ කටයුතු කිරීම**

ගේ දොර වැඩ කටයුතු තනිවම කිරීමට පුළුවන, කලාතුරකින් ඉතා අසීරු කාර්යයන් සඳහා වෙනත් අයගේ සහයෝගය අවශ්‍යය වේ. 1

ගේ දොර එදිනෙදා සිදු කරන සැහැල්ලු වැඩ කටයුතු තනිවම කළ හැක, උදා. පිඟන් කෝප්ප සේදීම, ඇඳන් අස් පස් කිරීම. 1

ගේ දොර එදිනෙදා සිදු කරන සැහැල්ලු වැඩ කටයුතු තනිවම කළ හැකි නමුත් එම වැඩ කටයුතු වල පිරිසිදු බව පිළිබඳව සෑහීමකට පත් විය නොහැක. 1

ගේ දොර සෑම වැඩ කටයුත්තක් සඳහාම වෙනත් අයගේ සහයෝගය අවශ්‍ය වේ.1

ගේ දොර එදිනෙදා කිසිම වැඩ කටයුත්තකට සහභාගී නොවේ. 0

E. **ඇඳුම් සෝදා ගැනීම**

තමන්ගේ සියලුම ඇඳුම් තනිවම සෝදා ගත හැක. 1

කුඩා ඇඳුමක් පමණක් තනිවම සෝදා ගත හැක, උදා. ලේන්සුවක්, කුඩා තුවායක් වැනි.

1

තමන්ගේ සියලුම ඇඳුම් වෙනත් අය විසින් සෝදා දිය යුතුයි. 0

F. **ගමන් බිමන් යාම**

පොදු ප්‍රවාහන සේවා භාවිතා කරමින් හෝ වාහනයක් පදවාගෙන තනිවම ගමන් බිමන් යා හැක. 1

කුලී රථයක තනිවම ගමන් බිමන් යා හැකි වුවත් පොදු ප්‍රවාහන සේවා භාවිතා කරමින් ගමන් බිමන් යා නොහැකිය. 1

තව කවුරුන්ගේ හෝ සහය ඇතිව පොදු ප්‍රවාහන සේවා භාවිතා කරමින් ගමන් බිමන් යා හැක. 1

තව කවුරුන් හෝ සමඟ කුලී රථයක ගමන් බිමන් යා හැක. 0

ගමන් බිමන් යාමට නොහැකිය. 0

G. **තමා ගත යුතු ඖෂධ තනිවම ගැනීමේ හැකියාව**

නියමිත වේලාවට නියමිත මාත්‍රාවට අනුව ඖෂධ තනිව ලබා ගත හැක. 1

නියමිත මාත්‍රාවට අනුව වෙන කවුරුන් හෝ කලින් ඖෂධ පිළියෙල කර තබා ඇත්නම් ඒවා ලබා ගත හැක. 0

තමා ගත යුතු ඖෂධ තනිවම ගැනීමට නොහැක. 0

H. මුදල් හැසිරවීමට ඇති හැකියාව

මුදල් සම්බන්ධ කාරණා තනිවම කළමනාකරණය කල හැක (උදා. විදුලි බිල්පත්, ජල බිල්පත් ආදිය ගෙවීම, බැංකුවට යාම) 1

එදිනෙදා අවශ්‍ය සුළු දේවල් මිලදී ගත හැකි නමුත් බැංකු කටයුතු වලදී හෝ විශාල මිලදී ගැනීම් වලදී උදව් අවශ්‍යය වේ. 1

මුදල් හැසිරවීමට නොහැක. 0

සම්පූර්ණ ලකුණ............

මෙම ප්‍රශ්නාවලිය මෙතැනින් අවසන්. ඔබ දැක්වූ සහයෝගයට ස්තූතියි.

Lawton MP, Brody EM; Assessment of Older People: Self-Maintaining and Instrumental Activities of Daily Living, The Gerontologist 1969; 9 (3_Part_1): 179–186, doi:10.1093/geront/9.3_Part_1.179. (Translated and) Reproduced by permission of Oxford University Press on behalf of The Gerontological Society of America. © 1969 The Gerontological Society of America. All rights reserved. For permissions please email journals.permissions@oup.com. Please visit: https://academic.oup.com/gerontologist/article/9/3_Part_1/179/552574

This scale is not included under the Creative Commons license of this publication.

Translation disclaimer:

“OUP and the GSA are not responsible or in any way liable for the accuracy of the translation. (Translator name) is solely responsible for the translation in this publication/reprint.”
