# Supplementary material for: Cross-cultural adaptation and psychometric evaluation of the Sinhala version of Lawton Instrumental Activities of Daily Living Scale
Source: PLoS One. 2018 Jun 28;13(6):e0199820. doi: 10.1371/journal.pone.0199820 (PMC6023108; doi:10.1371/journal.pone.0199820)
Supplement: S1 Fig — (PDF) [file pone.0199820.s003.pdf]

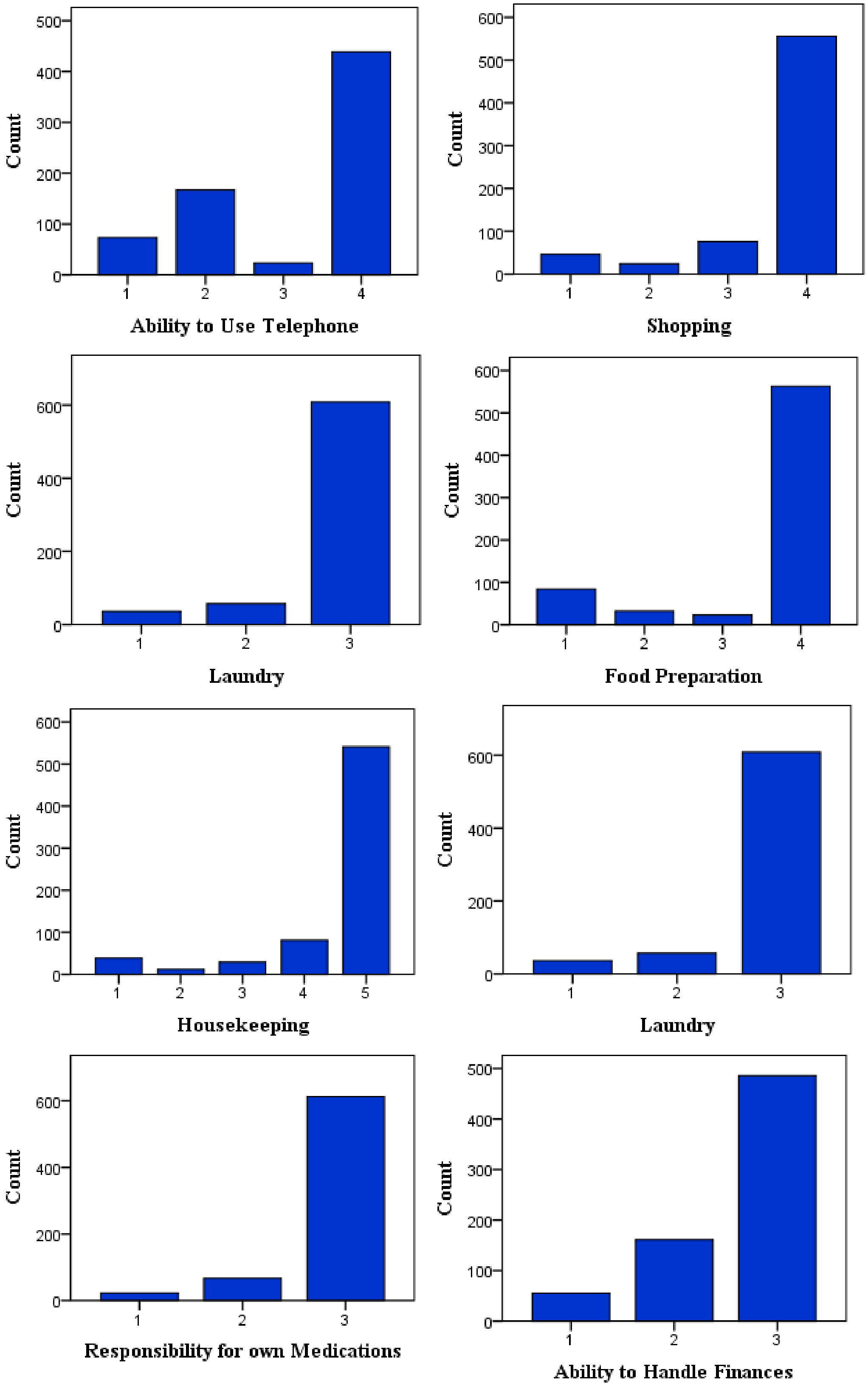

S1 Figure. The frequency distribution of the responses for each item of the Lawton IADL-Sinhala version.
