# Supplementary figures and images for: Cross-cultural adaptation and psychometric evaluation of the Sinhala version of Lawton Instrumental Activities of Daily Living Scale
Source: PLoS One. 2018 Jun 28;13(6):e0199820. doi: 10.1371/journal.pone.0199820 (PMC6023108; doi:10.1371/journal.pone.0199820)

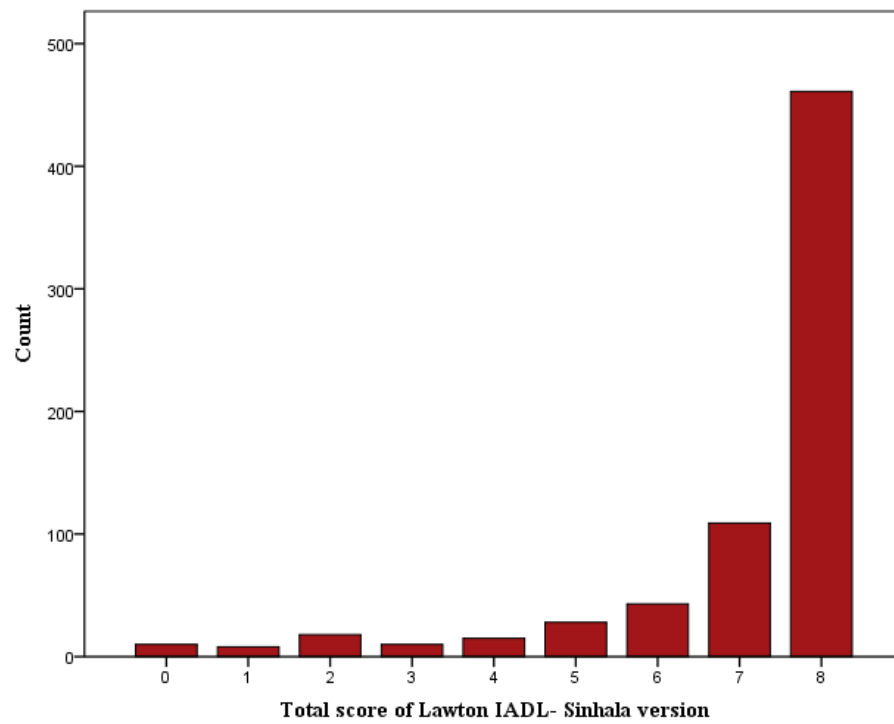

**S2 Figure. The frequency distribution of the overall Lawton IADL score-Sinhala version.**

Supplement: S2 Fig — (PDF) [file pone.0199820.s004.pdf]

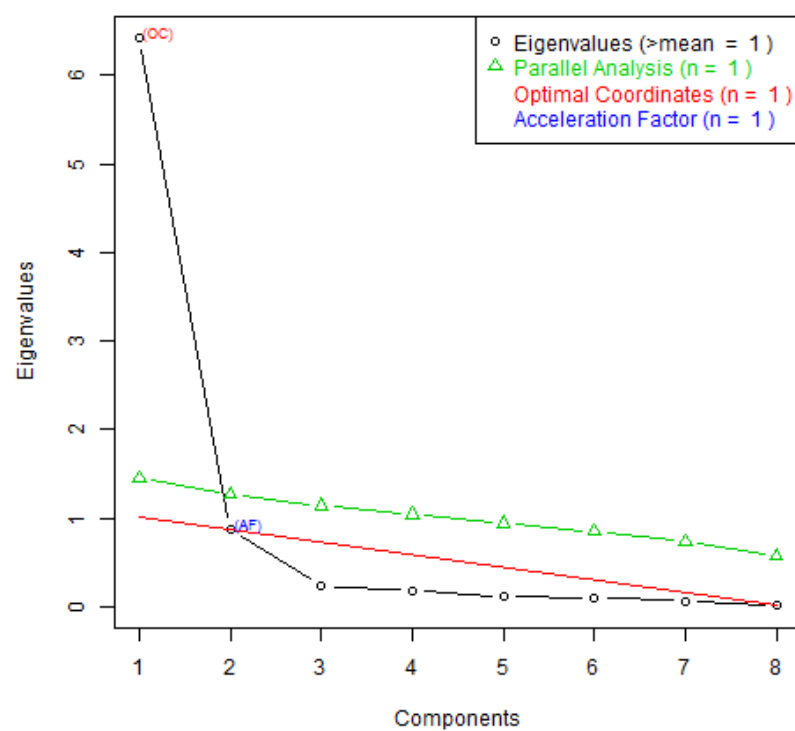

**S3 Figure. Parallel analysis results for females.**

Supplement: S3 Fig — (PDF) [file pone.0199820.s005.pdf]

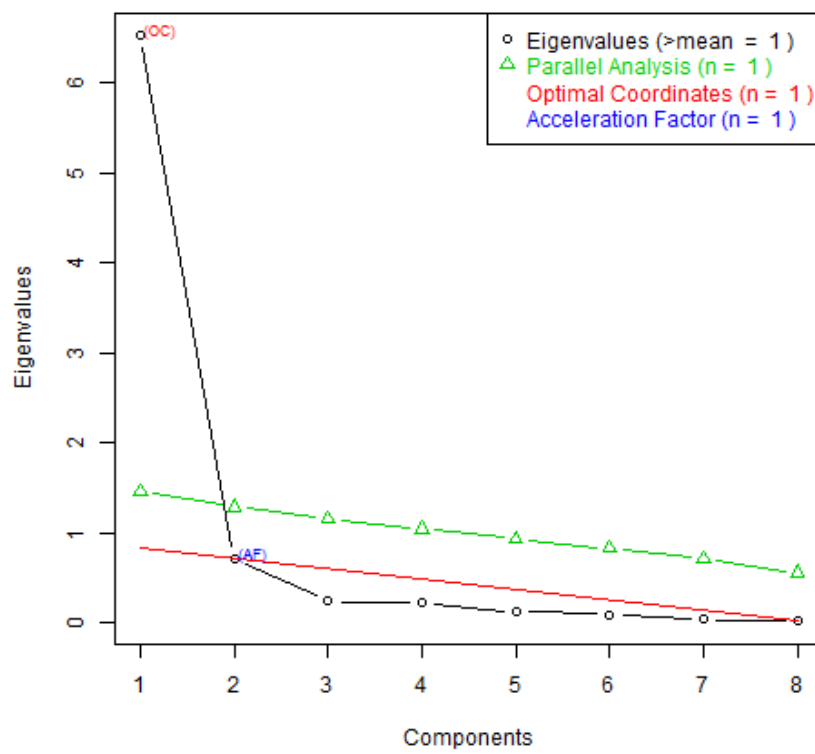

**S4 Figure. Parallel analysis results for males.**

Supplement: S4 Fig — (PDF) [file pone.0199820.s006.pdf]

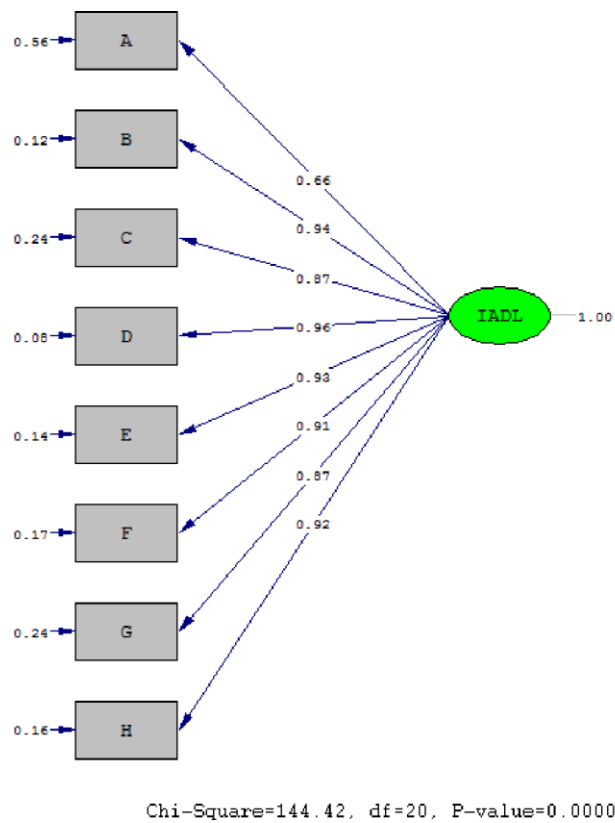

**S5 Figure. Confirmatory factor analysis model with standardized factor loadings.**

Supplement: S5 Fig — (PDF) [file pone.0199820.s007.pdf]
