# Supplementary material for: Cross-cultural adaptation and psychometric evaluation of the Sinhala version of Lawton Instrumental Activities of Daily Living Scale
Source: PLoS One. 2018 Jun 28;13(6):e0199820. doi: 10.1371/journal.pone.0199820 (PMC6023108; doi:10.1371/journal.pone.0199820)
Supplement: S1 Table — (PDF) [file pone.0199820.s008.pdf]

S1 Table. Item wise inter-rater reliability when original responses for Lawton IADL scale-Sinhala version coded as binary.

Item	Item description	PI-A (n=13)			PI-B (n=15)			PI-C (n=17)			PI-D (n=17)			PI-E (n=21)		
		**p**	***κ***	**Gwet's AC_1**	**p**	***κ***	**Gwet's AC_1**	**p**	***κ***	**Gwet's AC_1**	**p**	***κ***	**Gwet's AC_1**	**p**	***κ***	**Gwet's AC_1**
Item 1	Ability to use telephone	0.92	0.75	0.88		Not computed‡		1.00	1.00	1.00	0.94	0.76	0.91	0.95	**0.64**	0.94
Item 2	Shopping	0.92	0.75	0.88	0.80	**0.44**	0.68	0.76	0.46	0.59	1.00	1.00	1.00	0.90	**-0.05**	0.89
Item 3	Food preparation	0.92	0.75	0.88	0.66	**0.00**	0.53	0.94	0.86	0.89	0.82	**0.00**	0.78	0.95	**0.64**	0.94
Item 4	Housekeeping		Not computed‡		0.93	**0.00**	0.92	0.88	**0.00**	0.86	0.94	**0.00**	0.93		Not computed‡	
Item 5	Laundry		Not computed‡		0.93	**0.63**	0.91	1.00	1.00	1.00	0.94	**0.00**	0.93		Not computed‡	
Item 6	Mode of transportation		Not computed‡		0.93	0.76	0.90		Not computed‡		0.88	**0.43**	0.85		Not computed‡	
Item 7	Responsibility of own medication	1.00	1.00	1.00	1.00	1.00	1.00	1.00	1.00	1.00	1.00	1.00	1.00	1.00	1.00	1.00
Item 8	Ability to handle finances		Not computed‡		0.93	**0.00**	0.92	0.82	**-0.08**	0.78	1.00	1.00	1.00	0.95	**0.00**	0.95

p- Unweighted percentage agreement coefficient, ***κ*** - Cohen's kappa

Non-significant agreement coefficients (p>0.05) and zero agreement coefficients are displayed in bold.

Not computed‡, since ratings do not vary.
