# Supplementary material for: Cross-cultural adaptation and psychometric evaluation of the Sinhala version of Lawton Instrumental Activities of Daily Living Scale
Source: PLoS One. 2018 Jun 28;13(6):e0199820. doi: 10.1371/journal.pone.0199820 (PMC6023108; doi:10.1371/journal.pone.0199820)
Supplement: S2 Table — (PDF) [file pone.0199820.s009.pdf]

S2 Table. Polychoric (two step) correlation matrix used in EFA (entire sample).

	Item 1	Item 2	Item 3	Item 4	Item 5	Item 6	Item 7	Item 8
Item 1	1.000							
Item 2	.639	1.000						
Item 3	.433	.803	1.000					
Item 4	.541	.883	.868	1.000				
Item 5	.396	.819	.843	.925	1.000			
Item 6	.606	.863	.764	.862	.829	1.000		
Item 7	.632	.803	.792	.808	.771	.798	1.000	
Item 8	.717	.894	.672	.820	.750	.855	.819	1.000
