# Supplementary material for: Cross-cultural adaptation and psychometric evaluation of the Sinhala version of Lawton Instrumental Activities of Daily Living Scale
Source: PLoS One. 2018 Jun 28;13(6):e0199820. doi: 10.1371/journal.pone.0199820 (PMC6023108; doi:10.1371/journal.pone.0199820)
Supplement: S3 Table — (PDF) [file pone.0199820.s010.pdf]

S3 Table. Polychoric (two step) correlation matrix used in EFA for females.

	Item 1	Item 2	Item 3	Item 4	Item 5	Item 6	Item 7	Item 8
Item 1	1.000							
Item 2	.639	1.000						
Item 3	.510	.890	1.000					
Item 4	.503	.846	.930	1.000				
Item 5	.232	.779	.884	.892	1.000			
Item 6	.603	.838	.902	.871	.859	1.000		
Item 7	.628	.807	.850	.810	.743	.777	1.000	
Item 8	.701	.890	.852	.817	.711	.840	.813	1.000
