# Supplementary material for: Cross-cultural adaptation and psychometric evaluation of the Sinhala version of Lawton Instrumental Activities of Daily Living Scale
Source: PLoS One. 2018 Jun 28;13(6):e0199820. doi: 10.1371/journal.pone.0199820 (PMC6023108; doi:10.1371/journal.pone.0199820)
Supplement: S4 Table — (PDF) [file pone.0199820.s011.pdf]

S4 Table. Polychoric (two step) correlation matrix used in EFA for males.

	Item 1	Item 2	Item 3	Item 4	Item 5	Item 6	Item 7	Item 8
Item 1	1.000							
Item 2	.631	1.000						
Item 3	.421	.834	1.000					
Item 4	.573	.927	.883	1.000				
Item 5	.533	.883	.810	.955	1.000			
Item 6	.615	.896	.804	.885	.875	1.000		
Item 7	.653	.825	.759	.816	.792	.871	1.000	
Item 8	.742	.893	.641	.831	.818	.872	.854	1.000
