# Supplementary material for: Cross-cultural adaptation and psychometric evaluation of the Sinhala version of Lawton Instrumental Activities of Daily Living Scale
Source: PLoS One. 2018 Jun 28;13(6):e0199820. doi: 10.1371/journal.pone.0199820 (PMC6023108; doi:10.1371/journal.pone.0199820)
Supplement: S5 Table — (PDF) [file pone.0199820.s012.pdf]

S5 Table. Results of exploratory factor analysis by sex.

Items	Item description	Female* (n=377)			Male§ (n=325)		
		Communality	Factor loading	Item-scale correlation†	Communality	Factor loading	Item-scale correlation†
Item 1	Ability to use telephone	0.357	0.598	0.513	0.421	0.649	0.501
Item 2	Shopping	0.868	0.932	0.845	0.925	0.962	0.893
Item 3	Food preparation	0.934	0.966	0.837	0.691	0.831	0.683
Item 4	Housekeeping	0.877	0.936	0.848	0.923	0.961	0.916
Item 5	Laundry	0.713	0.844	0.705	0.853	0.923	0.838
Item 6	Mode of transportation	0.872	0.934	0.828	0.899	0.948	0.848
Item 7	Responsibility of own medication	0.774	0.880	0.719	0.804	0.897	0.774
Item 8	Ability to handle finances	0.837	0.915	0.831	0.828	0.910	0.826

*KMO=0.724, Bartlett's test ($p<0.001$), Fit indices: GFI (ULS) =0.978, RMSR =0.075

§KMO=0.870, Bartlett's test ($p<0.001$), Fit indices: GFI (ULS) =0.983, RMSR =0.056

†Item total correlation with its own Lawton IADL scale corrected for overlap.
