# Supplementary material for: Cross-cultural adaptation and psychometric evaluation of the Sinhala version of Lawton Instrumental Activities of Daily Living Scale
Source: PLoS One. 2018 Jun 28;13(6):e0199820. doi: 10.1371/journal.pone.0199820 (PMC6023108; doi:10.1371/journal.pone.0199820)
Supplement: S6 Table — (PDF) [file pone.0199820.s013.pdf]

S6 Table. Results of the confirmatory factor analysis by sex.

Items	Item description	Female (n=377)			Male (n=325)		
		Standardized factor loading	Standard error	t value	Standardized factor loading	Standard error	t value
Item 1	Ability to use telephone	0.645	0.044	14.663	0.673	0.052	13.044
Item 2	Shopping	0.924	0.018	51.231	0.950	0.016	59.897
Item 3	Food preparation	0.973	0.012	81.242	0.860	0.029	29.906
Item 4	Housekeeping	0.943	0.013	70.872	0.981	0.008	127.817
Item 5	Laundry	0.899	0.028	32.620	0.951	0.017	56.582
Item 6	Mode of transportation	0.923	0.016	57.708	0.936	0.018	52.322
Item 7	Responsibility of own medication	0.869	0.034	25.617	0.891	0.028	32.410
Item 8	Ability to handle finances	0.919	0.018	50.513	0.916	0.020	46.697
