# Supplementary material for: Cross-cultural adaptation and psychometric evaluation of the Sinhala version of Lawton Instrumental Activities of Daily Living Scale
Source: PLoS One. 2018 Jun 28;13(6):e0199820. doi: 10.1371/journal.pone.0199820 (PMC6023108; doi:10.1371/journal.pone.0199820)
Supplement: S7 Table — (PDF) [file pone.0199820.s014.pdf]

S7 Table. Goodness of fit indices of confirmatory factor analysis by sex.

	χ^2	df	χ^2/df	p	RMSEA (90% CI)	NNFI	CFI	SRMR	PNFI
Female	116.02	20	5.80	<0.001	0.379 (0.360-0.398)	0.972	0.980	0.073	0.697
Male	72.28	20	3.61	<0.001	0.326 (0.306-0.347)	0.982	0.987	0.054	0.702
